# Establish a Digital Real-Time Learning System With Push Notifications

**DOI:** 10.3389/fpsyg.2022.767389

**Published:** 2022-02-18

**Authors:** Hsin-Te Wu

**Affiliations:** Department of Computer Science and Information Engineering, National Ilan University, Yilan County, Taiwan

**Keywords:** e-learning, push notifications, real-time learning, problem-based learning, social network

## Abstract

This study proposes a push notification system that combines digital real-time learning, roll-call, and feedback collection functions. With the gradually flourishing online real-time learning systems, this research further builds roll-call and feedback functions for students to enhance concentration and provide opinions. Additionally, the lecturers can do a roll call irregularly and randomly through the push notification function, avoiding students logging in but away from the keyboard. Lecturers can also send questions to a specific student or invite all students to answer; the replies can show students' learning performance. The system will store each notification in a database and analyse messages automatically to record roll calls. Moreover, the system can record the time and intervals of student feedback, enabling lecturers to check students' attention and learning conditions. Currently, most online digital systems depend on the lecturer to be responsible for the entire system; taking a roll call and asking questions will consume the lecturer's teaching time and strength. The system developed in this article can do roll calls and feedback by push notifications, reducing lecturers' workload. Furthermore, the roll-call and automatic record functions can save the time of paperwork after a course.

## 1. Introduction

The severe impact of the epidemic has forced many in-person classes to transfer into online courses; however, without teaching assistants, lecturers have to be in charge of teaching materials, software and hardware, and roll calls, which causes heavy workloads. E-learning systems enable students to learn anytime and anywhere; teachers can also provide one-on-one instruction to enhance learning quality (Kekkonen-Moneta and Moneta, 2002; Ruiz et al., 2006). Nonetheless, online courses have increased rapidly during the COVID-19 pandemic, leading to worse learning quality (Agarwal et al., 2021), and many online teaching systems do not offer one-on-one instruction functions. Consequently, although students logged in to the e-learning platform, they might be doing other matters or taking rests; such a situation will reduce learning motivation and have poor performance. Additionally, most online courses have more than 13 students, which contradicts the best student-teacher ratio of 13:1 (Muñoz-Merino et al., 2021), generating worse learning quality and heavier teaching workloads. E-learning intends to provide students with learning environments anytime and anywhere and enable them to deliver teaching feedback to teachers through online tests and appropriate methods, making lecturers take care of students' learning conditions with one-on-one instruction. Currently, many free digital platforms can offer online teaching functions; yet, the simplified features fail to provide inter-actions (Wu et al., 2020); therefore, students may feel bored and have lower learning motivation. As a result, an inter-active system to deliver student feedback and opinions is essential.

Literature (Llamas-Nistal et al., 2011) has suggested that an e-learning platform with more inter-active functions will benefit learning; yet, teachers should familiarize themselves with the operation to ensure learning performance. In Literature (Almaiah and Alyoussef, 2019), the study has pointed out that a platform only offers a learning medium; teachers should design teaching materials and homework in advance. Unlike in-person courses, tutors should design online materials and prepare the teaching approaches before each class to achieve optimal online teaching. The research in Literature (Wang and Liu, 2008) conducts electronic experiment teaching through cameras, which requires equipment upgrade for students to see the entire experimental process. Literature (Dodero et al., 2014) further mentions that e-learning platforms should be modularized for users to choose suitable functions. Meanwhile, Literature (Maher et al., 2020) has mentioned the higher flexibility of e-learning than in-person courses; however, teachers will need to rearrange the course design and materials. Finally, Literature (Baehr, 2012) explains that an e-learning system needs to have feedback functions and one-on-one learning incentives to boost overall learning performance. The system in our research utilizes a free-of-charge e-learning platform but adds auxiliary tools to improve the learning rate. Moreover, apart from understanding the concentration condition of students, the push notification function can prevent students from logging in but staying away from the keyboard; the system can do roll calls irregularly, helping teachers to focus on teaching while maintaining the attendance rate. Although many synchronous learning platforms are free, the limited functions only allow the system to perform online teaching. To enhance teaching quality, the method proposed in this article can conduct digital roll calls to stop students from staying away from the keyboard or lowering their concentration. Additionally, this system helps teachers record attendance without using pen and paper.

Based on the above issue, this article proposes a digital real-time learning system with push notifications. During the pandemic, many tutors teach by online software such as Google Meet or Microsoft Teams; nevertheless, the software usually only provides simplified synchronous learning without any inter-active tools for lecturers to use. This approach designed in our study has a push notification function, enabling teachers to send questions to students regularly, and the system will record the details of the questions and replies in the database. Furthermore, the system can do roll calls according to the student information and detect the response time and the reply numbers to a message. Thus, the developed system can reduce lecturers' workloads by eliminating the additional effort to pay attention to students' conditions, allowing teachers to concentrate on the course; the system can also improve concentration and calculate the attendance and learning rate, which becomes an auxiliary tool for teaching to enrich learning quality. The key contributions of our study are: (1). Use the push notification function to implement random roll calls; (2). Send course questions and answers *via* the push notification function; (3). The system will record every information in the database and automatically analyze the roll call messages; and (4). The system can reduce teachers' paperwork. The primary contribution of this article is the capability to synchronize online software with its push notification system freely to enhance online teaching quality. With the limited functions of free online teaching platforms, teachers need to do a roll call by the conventional method, read aloud the names of the students one by one to check their attendance, which consumes the course time. The proposed system in this study can implement a roll call randomly and pick any student to answer questions. Moreover, different from other push notification systems on the market, the developed system can concatenate the roll call mechanism and set the function randomly and automatically through the push notification; yet, the push notification function on other systems can only send messages to interact.

## 2. Related Works

Literature (Chen et al., 2018) primarily proposes a new model to explore users' inter-actions and the willingness to continue the discovery in Massive Open Online Courses (MOOCs). Literature (Hsu et al., 2018) further surveys a comparison about users' willingness to learn between MOOCs and traditional digital learning platforms. The research in Literature (Chang et al., 2019) mentions that virtualized MOOCs tend to cause distraction; therefore, using electroencephalography analysis to find out that students' learning performance in MOOCs is significantly superior to that of traditional PowerPoint-material teaching. The research in Literature (Chen and Hsiao-FenTseng, 2012) points out that it requires considering teachers' teaching methods on the platform and the preparation of teaching materials in digital learning. While maintaining students' concentration is critical in digital learning, the study in Literature (Chen, 2012) introduces a system to capture students' facial expressions by the camera, judging whether they are still paying attention to the class. Additionally, Literature (Hwang et al., 2021) reveals that most research has compared the learning performance between traditional textbook teaching and the teaching using electronic book readers in the classroom. In that article, the study investigates the use of writable electronic book readers in the English class in an elementary school and examines the learning results and achievements of the sharing mechanism with in-class and out-of-classroom notes; the findings have shown that a sharing electronic book reader with notes can benefit students' learning results. Meanwhile, Literature (Hwang et al., 2021) also mentions that the article number regarding MOOC research has been increasing in recent years after it becomes a mainstream method since 2011, and most research focuses on resolving the issues in self-learning. Literature (Alzahrani and Meccawy, 2021) further explores why MOOCs becomes popular among adult learners. The study discovers that many professionals utilize MOOCs for their career development as there are plentiful inter-disciplinary courses. Literature (Wu and Li, 2020) points out that the Electronic Word of Mouth (eWOM) is an important information source for learners in MOOC; nonetheless, little research discussed this factor. The new problem of learning resource mention identification in MOOC forums is mentioned in An et al. (2019), i.e., identifying resource mentions in discussions and classifying them into predefined resource types. The study in Literature (Rohan et al., 2021) suggests and evaluates a theoretic model to identify the factors that influence learners to continue participating in MOOCs; it is an expectation-confirmation model with a gamified structure and additional motivation.

Literature (Ikhsan et al., 2019) offers an investigation model to understand students' online learning performance and satisfaction. Since online learning is considered a useful learning tool; to reinforce students' performance, Literature (Tzu-Chi, 2020) introduces many strategies to implement in online-learning environments and guide students. Particularly, these strategies are useful in participant observation and self-regulated learning. Literature (Murad et al., 2020) aims to confirm the preparation of the school, teachers, and students and their conditions during the learning process while maintaining the teaching quality, user satisfaction, and both teachers' and students' learning performance. Moreover, Literature (Wang et al., 2021) reveals that the advance of MOOCs has successfully generated another subversive learning method called blended learning. The existing research has shown that blended learning is not only accepted widely by university students but also well-liked. Compared to traditional teaching approaches, blended learning has transferred the relationship between teaching and learning; hence, the assessment criterion should be amended accordingly. Literature (Saliah-Hassane et al., 2019) defines the storage, retrieval, and access to online laboratory methods for intelligent inter-active students. The discussion in Literature (Tao et al., 2014) indicates that most of the existing research of adaptive online learning systems primarily focuses on exploring students' pre- and post-learning performance but ignores discussing the suitable teaching styles and the selected multi-media. Literature (Xing et al., 2021) also proposes a new predictive modeling approach to enhance the model portability for different classes in one course as time goes. Finally, Literature (Zel et al., 2021) states how facial recognition technology can create a fascinating learning experience for different online participants.

## 3. Method

### 3.1. System Model

When teachers utilize the free online teaching software on the market, the platforms usually only provide online teaching functions; thus, the teacher still needs to do roll calls and assign homework in person. In this article, we propose a system with a push notification function for regular roll calls so that students can respond through push notifications, and teachers can thus check if students are paying attention. Furthermore, teachers can ask questions *via* the push notification function, and the system will detect the student's answering time and check the correctness. Meanwhile, the system will also record the number of times each student answers. This developed system aims to promote online teaching quality and analyze students' learning performance and whether they are paying attention. In recent years, due to the severe epidemic of COVID-19, many teachers have changed to teach online; using the developed system can improve students' learning performance.

**Figure 1:**
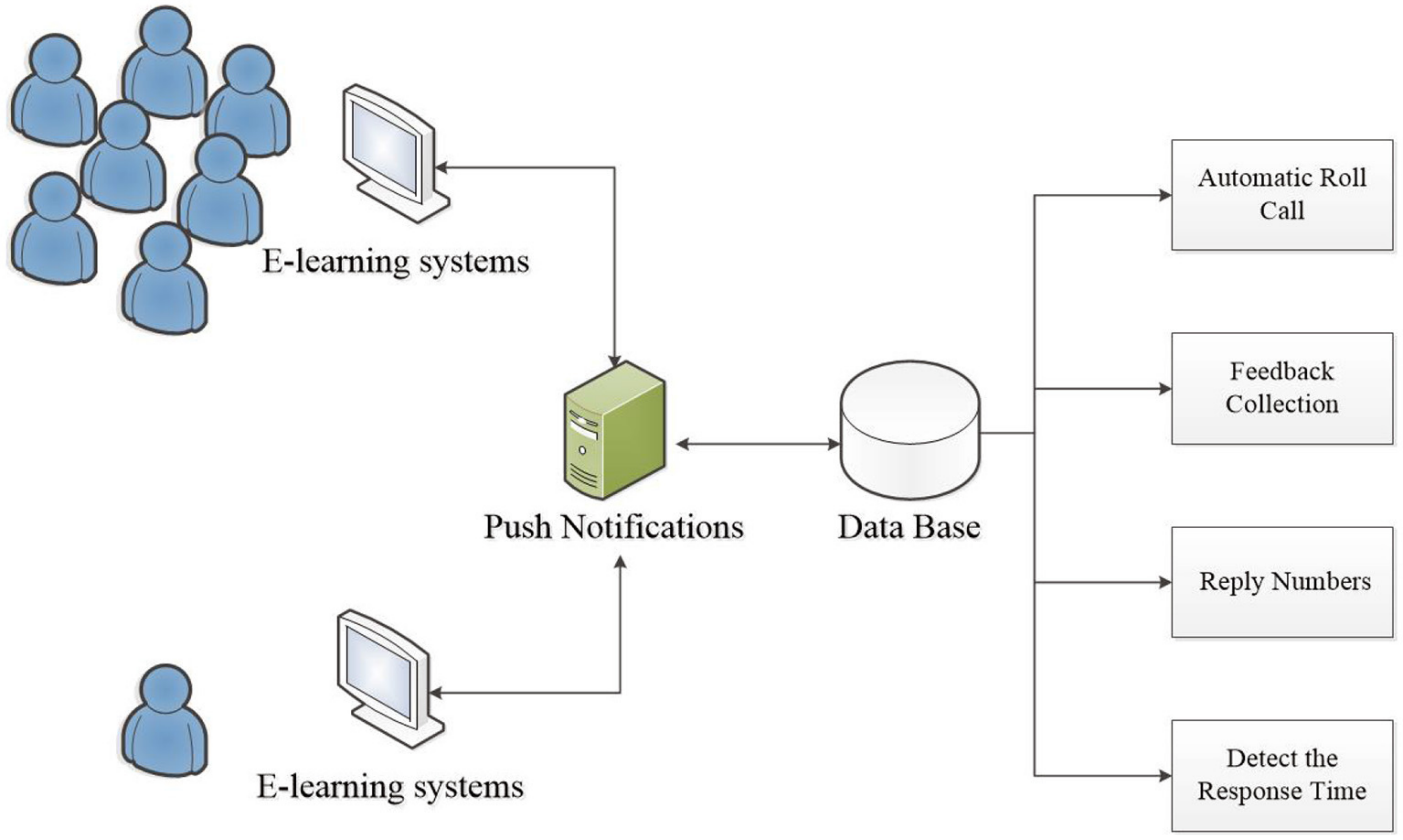
System model.

The system design of automatic analysis is demonstrated in Figure 1. Our system combines a push notification function and an Artificial Intelligence (AI) analysis module; when the teacher sends a push notification to ask a question, students can also answer *via* push notification. Apart from detecting the time intervals that a student responds, the system will also judge the correctness of the answer. Moreover, except for checking the correctness of the answers, if the teacher sets answers and defines questions scores in advance, the system will automatically mark scores and save the results in the database. Finally, the system will analyze students learning conditions and reveal students' overall scores to the teacher. To be more specific, the system calculates students' time intervals when they respond to a roll call or question, judging students' level of concentration and finding out if they logged in but stayed away from the keyboard; the automatic analysis mainly reports students' learning conditions.

### 3.2. The Proposed Scheme

**Figure 2:**
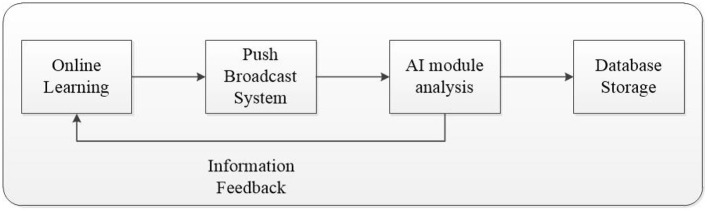
System model.

The schematic diagram of our system is shown in Figure 2. The symbols in this article are listed in Table 1. Using Google Meet or Microsoft Teams as the e-learning platform, lecturers can ask questions by the push notification function. In this designed system, push notifications will send by the LINE freeware application, and students can answer the questions *via* the system's web page after receiving the message. Afterward, the system will analyze the response time automatically. When the response intervals are too long, it might mean that students have a lower concentration or are absent. The interval detection formula is as below:


(1) $$\begin{array}{r} Count=\left\{\begin{array}{c} Count+1,\left(Q_{i}-Ans_{i,j}\right)>Threshold\\ Count,otherwise \end{array}\right\} \end{array}$$

*Q*_*i*_ represents the question sent time, *Ans*_*i, j*_ means the answering time of each student, and the *Threshold* is the threshold value of the interval. Formula (1) calculates the response time and how many students have exceeded the threshold value. As a result, the system will show the Count value to the teacher for exploring how many students have lowered their learning concentration, which helps the teacher judge to take a break or handle this situation by appropriate methods. Moreover, the system can do roll calls automatically to check the absent or late conditions. Tutors can set the absent rules in the system; for example, if the response time of a roll call is over twenty min, the system will mark the student as absent; if the response time of a roll call is over ten minutes but less than twenty min, the system will mark the student as late to the class.

**Table 1:** Summarizes of notations and symbols.

**Symbol**	**Meaning**
*Count*	Number of responses to messages
*Q*_*i*_	The question sent time
*Ans*_*i, j*_	The answering time of each student
*Threshold*	The threshold value of the interval
*RCA*_*i, j*_	The number of answering questions

Meanwhile, the system will automatically check the number of answering questions and show teachers the results to understand students' learning willingness toward the course. The statistical formula is as the following:


(2) $$\begin{array}{r} RCA_{i,j}=\left\{\begin{array}{c} RCA_{i,j}+1,\left(Q_{i}-Ans_{i,j}\right)<Threshold\\ RCA_{i,j},Ans_{i,j}=Null\\ RCA_{i,j},otherwise \end{array}\right\} \end{array}$$

*RCA*_*i, j*_ means the number of answering questions by each student. In the formula, if *Ans*_*i, j*_ is Null, the figure will not be included. The formula (2) generates the number of answering questions; if *Ans*_*i, j*_ is not Null, the calculation will eliminate the value with the answering interval and accumulate the value when it is smaller than the threshold.

The impact of the epidemic has caused many schools to transfer in-person teaching into online courses in several countries; nonetheless, it requires teachers to take care of students' learning conditions and opinions by employing more tools for obtaining learning feedback and performance. The developed system in this article utilizes the push notification function to ask questions and attain student feedback. The system can analyze the response intervals automatically. On the other hand, the roll-call function can reduce teachers' workloads. By sending questions irregularly through the system, the teaching can increase students' concentration. Furthermore, the suggested module is an additional system, which is compatible with various free online software.

## 4. Results

**Figure 3:**
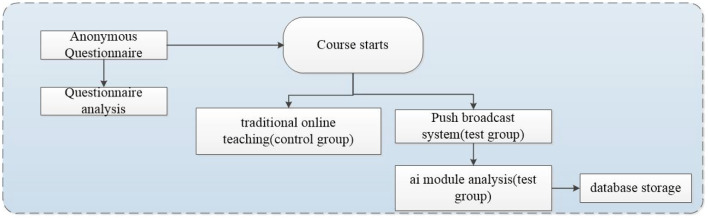
Experimental analysis process.

This study uses an anonymous questionnaire to evaluate users' satisfaction with the system and the teaching quality. Before operating the system, our team conducted an orientation to five teachers, hoping to familiarize them with the system and avoid the dislike caused by a lack of information adaptability. Figure 3 illustrates the experimental flow. Firstly, the five teachers tested the systems in the control and experiment groups. Next, these five teachers would choose two courses for the experiment; one course used a traditional online teaching system while the other used the real-time online system with a push notification function. Finally, after teaching, the teachers would complete an anonymous questionnaire to evaluate the results. Consequently, the experiment result has proved that the suggested system we designed can benefit the quality of online teaching, prevent students from logging in but staying away afterward, and reduce the administrative workload for teachers.

**Figure 4:**
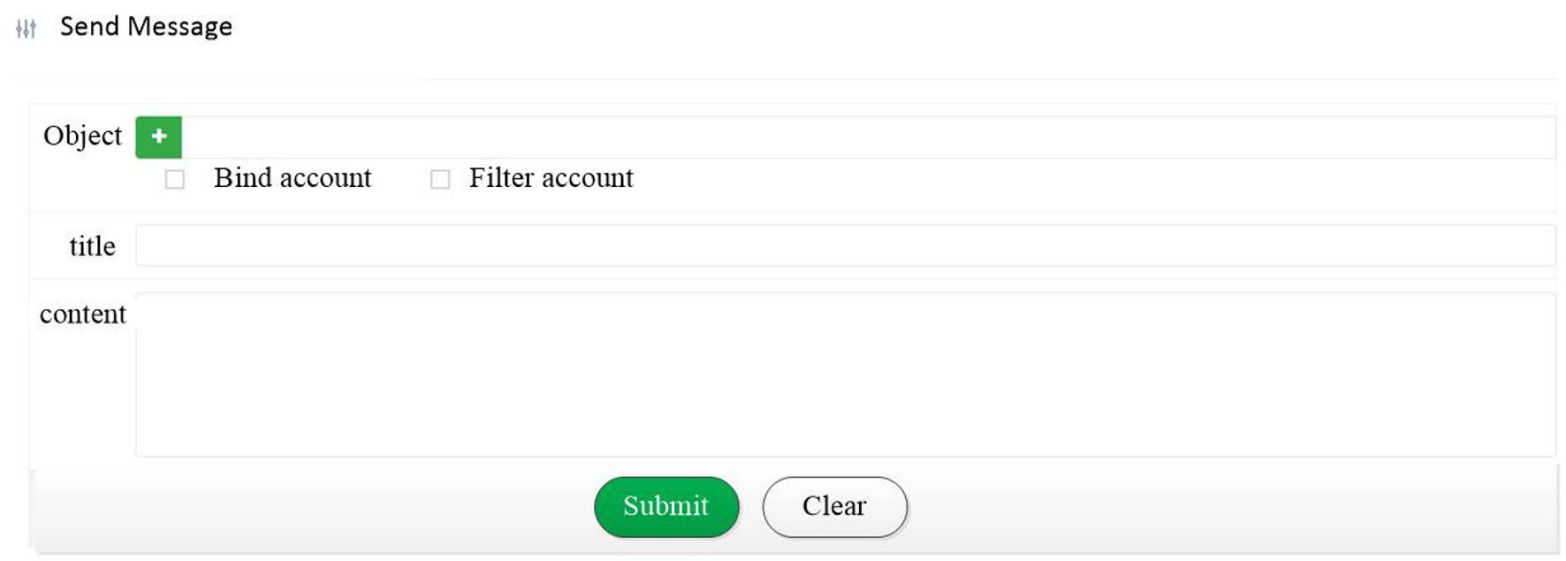
Push notification.

**Figure 5:**
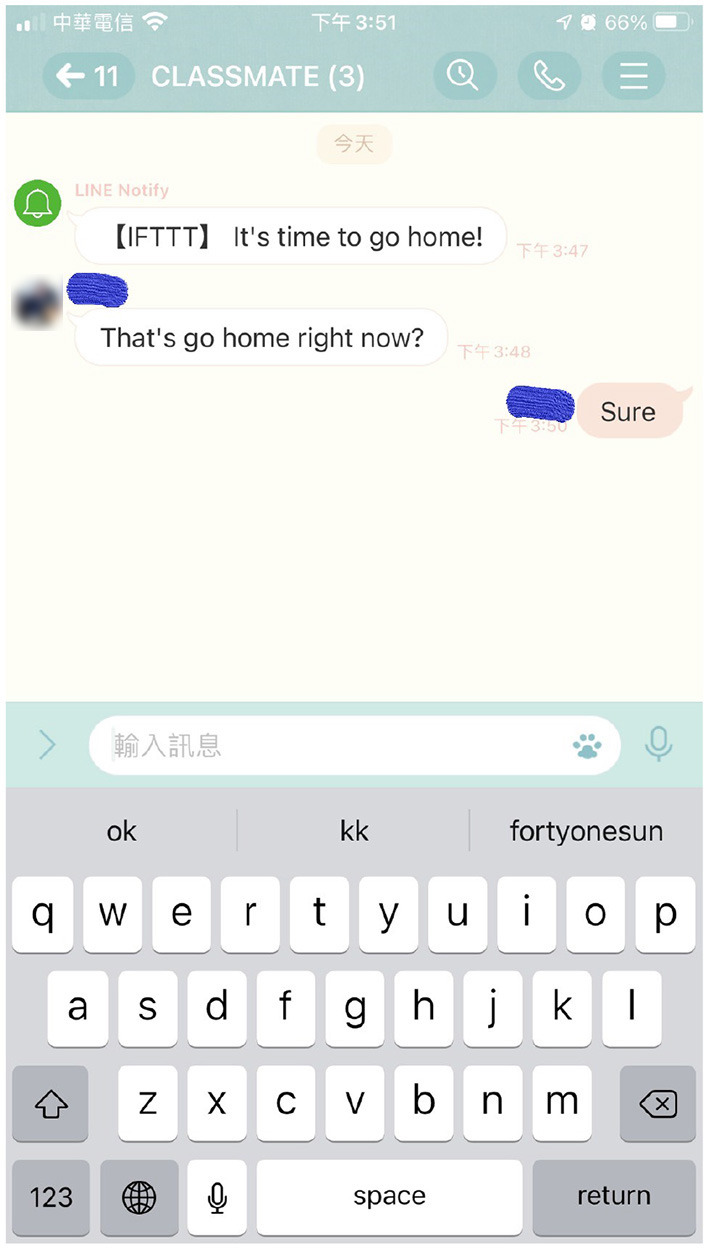
LINE push notification.

Our study has established a push-notification system that combines web pages to send teaching questions and collect feedback. The push notifications are sent by the LINE freeware application, and students can answer the questions *via* the system web page. As shown in Figure 4, the system can send group notifications, and students can reply directly on the web page. Moreover, the system has equipped with a database for storing and analyzing relevant information automatically. Figure 5 demonstrates a picture of when students received a LINE push notification. With the high penetration of the LINE freeware application, students tend to be more familiar with using LINE; consequently, sending messages through LINE can reduce the challenges of information adaptability, helping students to be familiar with the system rapidly. Table 2 demonstrates the hardware and software equipment used in the system. The research has invited teachers to use the system anonymously, and the teachers have found the system indeed improved students' concentration and inter-active levels. The result has shown that the proposed system can improve the performance of simplified digital learning platforms and enhance the teaching quality. This article conducts an anonymous questionnaire regarding the system performance; as a result, five teachers in total had responded to the questionnaire, as shown in Table 3. The results in the table reveal that the overall system is beneficial to teachers in various parts, such as the roll call process and the attendance rate. The study proposes a system that can be combined with simplified online-teaching systems, enhancing students' learning quality and lowering the situations students log in but stay away from the keyboard. The system functions compared with Literature (Wang and Liu, 2008) and (Dodero et al., 2014) are shown in Table 4. From the experimental results, the proposed system equips full functions that can benefit online teaching quality.

**Table 2:** Development tools.

**Hardware development tools**	**Software development tools**
Server	Windows 10
Laptop	PHP
	Dreamweaver

**Table 3:** Effectiveness analysis.

**Evaluation scale/item**	**Strongly**	**Agree**	**Neutral**	**Disagree**	**Strongly**
	**agree**				**disagree**
The system has improved					
the attendance rate.	1	4	0	0	0
The system has improved					
students' concentration.	3	2	0	0	0
The system has increased					
students' learning performance.	1	4	0	0	0
The system has					
lowered teachers' paperwork.	3	1	1	0	0
The system is not					
helpful to online teaching tools.	0	0	0	3	2
The system can					
assist in roll calls.	3	2	0	0	0
The system can					
enhance online teaching quality.	3	2	0	0	0

**Table 4:** Method comparison.

**Property / Method**	**Rohan et al. (2021)**	**Tao et al. (2014)**	**The proposed system**
Automatic Roll Call Function	Yes	Yes	Yes
Random Roll Call	No	No	Yes
Quiz Function	Yes	Yes	Yes
Messages	Yes	Yes	Yes
Student Engagement Analysis	Yes	Yes	Yes

## 5. Discussion

As the current pandemic transferring the original teaching methods globally, many students have to attend online courses instead of in-person classes. With the huge change and higher course options, making sure students' learning effectiveness and prevent them from lowering concentration have become a critical issue. In this article, we have established a push-notification digital real-time learning system, assisting teachers in conducting relevant works, such as roll calls, concentration analysis, and collecting learning feedback. The system can enhance students' concentration, preventing them from logging in but stay away from the keyboard. Our research also analyzes the response intervals by each student to judge if they have lowered attention; as a result, tutors can implement relevant actions according to the analysis to re-engage with their students and improve teaching quality. In the future, we aim to expand the system constantly. For instance, use deep learning to detect if students have turned off their screens or develop facial recognition to check if students have asked someone else to attend the class. We expect the suggested system can boost the course quality of e-learning, making the system universal and simple to reduce the challenges of information adaptability. The key of our system is the push notification function; however, too many expansion modules in a system will make the system too complicated and cause too much workload for teachers. While the system in this research is independent; therefore, teachers need to learn how to operate not only the online teaching platform but also the system we developed.

## Data Availability Statement

The original contributions presented in the study are included in the article/supplementary material, further inquiries can be directed to the corresponding author/s.

## Author Contributions

The author confirms contribution to this article as follows. HW: study conception and design, analysis and interpretation of results, and draft manuscript preparation. The author reviewed the results and approved the final version of the manuscript.

## Funding

This work was supported in part by the Ministry of Science and Technology of Taiwan, R.O.C., under Contracts MOST 109-2622-E-197-012 and MOST 110-2622-E-197-015. This research received no specific grant from any funding agency in the public, commercial, or not-for-profit sectors.

## Conflict of Interest

The author declares that the research was conducted in the absence of any commercial or financial relationships that could be construed as a potential conflict of interest.

## Publisher's Note

All claims expressed in this article are solely those of the authors and do not necessarily represent those of their affiliated organizations, or those of the publisher, the editors and the reviewers. Any product that may be evaluated in this article, or claim that may be made by its manufacturer, is not guaranteed or endorsed by the publisher.
